# Dietary patterns of U.S. older adults and their associations with diet quality, health and food insecurity

**DOI:** 10.1017/S1368980026102560

**Published:** 2026-04-30

**Authors:** AnnieBelle J. Sassine, Edwina A. Wambogo, Alanna Moshfegh, Nadine R. Sahyoun

**Affiliations:** 1 Nutrition and Food Science, University of Maryland at College Park, College of Agriculture and Natural Resources, USA; 2 National Cancer Institute, National Institute of Health, USA; 3 Food Surveys Research Group, US Department of Agriculture, USA

**Keywords:** Dietary patterns, Older adults, Food insecurity, Healthy Eating Index, Physical food insecurity

## Abstract

**Objective:**

Identify dietary patterns among U.S. older adults and examine their associations with sociodemographic and health characteristics including economic and physical functioning-related food insecurity.

**Design:**

Secondary analysis of dietary intake data from the 2013–2018 What We Eat in America component of NHANES. Dietary patterns were derived using cluster analysis and compared by diet quality (Healthy Eating Index (HEI)-2020), demographic characteristics, health indicators and multidimensional food insecurity.

**Setting:**

United States.

**Participants:**

A nationally representative sample of 5062 adults aged ≥ 60 years

**Results:**

Five dietary patterns were identified. The largest pattern, ‘juices, smoothies, grain drinks and soups’ (53·0 %), was characterised by the lowest mean energy and protein intake and a moderate HEI-2020 score (66·0 ± 0·8). In contrast, the ‘cooked cereals and yogurt’ pattern (10·8 %) had the highest HEI-2020 score (72·3 ± 1·4) and more favourable health indicators. Patterns high in processed meats and baked goods – ‘cured meats, sandwiches and sweet bakery products’ (18·1 %) and ‘meats, alcohol and quick breads’ (11·0 %) – had the lowest diet quality scores (48·5 ± 1·0 and 58·3 ± 1·6, respectively) and were more common among younger older adults, males, current smokers and individuals with obesity. Food insecurity due to both economic constraints and physical functioning limitations was most prevalent in the lower-quality soft-food pattern and least prevalent in the ‘Seafood and vegetables’ pattern (0·5 %).

**Conclusions:**

Distinct dietary patterns exist among U.S. older adults, with substantial variation in diet quality, health characteristics and food insecurity. Interventions should address both economic and functional barriers to support nutrient-dense, texture-appropriate diets in older adults.

As life expectancy increases, population aging is becoming a major societal trend^(1,2)^. Yet, longevity does not always equate with extended health span, with poor health in later years affecting quality of life and increasing healthcare costs^(3)^. According to the Global Burden of Disease Study (2019), six of the top fifteen risk factors for early deaths in the USA were related to diet: low intake of whole grains and legumes, and high intake of red meat, trans-fat, processed meat and sodium^(4)^. Research shows that dietary patterns play an important role in cognitive function, chronic diseases and mortality in older adults^(5–7)^, with healthy diets – rich in fruits, vegetables, whole grains, lean proteins and low-fat dairy – linked to better nutrition and longer life expectancy^(7,8)^.

A range of factors – social, cultural, physiological, psychological and financial – can shape the dietary choices of older adults^(9)^. The coexisting burden of poverty, medication expenses and food insecurity can limit access to nutritious foods^(9–11)^, while physical functioning limitations and chronic conditions may hinder food preparation and intake^(12–14)^. These barriers, along with depression, loneliness and medical or dental issues, contribute to inadequate dietary intake and reduced dietary diversity^(15–17)^, increasing the risk of malnutrition among older adults, particularly those with disabilities^(18–20)^. While prior research has identified dietary patterns in older populations, few studies have concurrently examined how food insecurity due to both economic constraints and physical functioning limitations relate to those patterns. This study fills that gap by integrating diet quality measures and multidimensional food access indicators.

A recent study of U.S. adults aged 65 and older revealed a significant decline in diet quality from 2001 to 2018, with over 60 % of older US adults having poor diet quality^(21)^. As the older adult population grows, addressing their nutritional needs becomes increasingly urgent, emphasising the need for research using robust dietary data. While most research assesses diet through quality indices or nutrient intake^(22–25)^, studies exploring dietary patterns in this group remain scarce. Dietary pattern analysis has emerged as a reliable approach in understanding the overall diet of a population, as it accounts for the combined effects of individual nutrients and foods^(26,27)^. Dietary patterns can be examined in several ways including the *a priori* method, which scores the quality of the diet^(26)^ and the *a posteriori* method which is an exploratory approach using existing dietary data to derive dietary patterns^(26,28)^. These dietary patterns can then be examined in association with other factors^(26,27)^. Understanding the factors associated with certain dietary habits may provide guidance in developing tailored nutrition education messages relevant to different older adult populations.

This paper aims to derive dietary patterns of older adults (60 years and above) in the United States using cluster analysis applied to dietary data from What We Eat in America (WWEIA), the dietary intake component of the National Health and Nutrition Examination Survey (NHANES) 2013–2018. The study also explores how these patterns relate to sociodemographic characteristics, food security, health status and diet quality.

The primary goals of our study were to (1) derive dietary patterns among adults aged 60 years and older, who represent a population vulnerable to chronic diseases; (2) examine sociodemographic and health-related characteristics across dietary patterns to guide targeted nutrition interventions; and (3) assess how food insecurity – both economic and physical functioning limitations – is associated with dietary choices.

## Methodology

### Study population and data source

This study analyses data from 5062 adults aged 60 years and older who participated in the 2013–2018 WWEIA, NHANES surveys. NHANES is a nationally representative, multistage probability sample of the civilian, noninstitutionalised U.S. population, administered by the National Center for Health Statistics (NCHS). To improve estimate reliability, NHANES oversamples older adults defined in NHANES as aged 60 years and over. Participants completed home interviews via the Computer-Assisted Personal Interview system^(29–31)^, followed by a health exam and a 24-hour dietary recall at the Mobile Examination Clinic. The first (Day 1) 24-hour dietary recall is conducted face-to-face using the USDA’s Automated Multiple-Pass Method to gather details about the type and quantity of all foods and beverages consumed during the day before the recall^(32)^. The second (Day 2) 24-hour dietary recall interview is conducted by telephone three to 10 d later. For this analysis, only reliable Day 1 recalls were used to generate dietary patterns, consistent with recommendations from the National Cancer Institute for estimating population mean intake^(33)^. Exclusions included participants without dietary recalls (2013–2014 *n* 199, 2015–2016 *n* 103, 2017–2018 *n* 189) or with unreliable recalls (2013–2014 *n* 19, 2015–2016 *n* 22, 2017–2018 *n* 18). Other exclusions include marital status (*n* 4), living arrangement (*n* 2), educational level (*n* 10), poverty-to-income ratio (*n* 545), BMI (*n* 82), smoking status (*n* 8), self-reported hypertension (*n* 11), self-reported diabetes (*n* 1), self-reported stroke (*n* 12), significant weight loss (*n* 123), self-reported health (*n* 127) and self-reported diet quality (*n* 2). The criteria and methods for dietary data quality control are described in detail elsewhere^(34–36)^. All NHANES survey protocols are approved by the NCHS Research Ethics Review Board, and written informed consent is obtained from all participants by NCHS. Because NHANES data are fully de-identified before public release, the present study was classified as non-human-subjects research and did not require additional IRB review.

### Sociodemographic and economic variables

Sociodemographic information was collected using the demographics questionnaire of NHANES, which was administered in the respondents’ homes. Age was categorised into two groups: 60–69 and 70 years and older. Race was classified as non-Hispanic White, non-Hispanic Black, Hispanic (including Mexicans) and Other. Due to small sample sizes, the other category combined Non-Hispanic Asians, individuals of other racial/ethnic backgrounds and those who reported multiple races.

Other sociodemographic characteristics included sex, education (below high school, high school or above) and marital status (married or living with a partner *v*. divorced, separated or never married). Living arrangement categories (alone, with spouse or with others) were based on household size and marital status. Income was categorised using the federal poverty level: ≤ 185 % *v*. > 185 %, determined by the poverty-to-income ratio. A poverty-to-income ratio value ≤ 1·85 indicated poverty and is commonly used to determine eligibility for federal food and nutrition assistance programmes^(37)^.

### Health-related variables

Information on BMI was collected in the Mobile Examination Clinic by trained health technicians and categorised as follows: older adults who are underweight (BMI < 18·5 kg/m^2^), of normal weight (BMI 18·5–24·9 kg/m^2^), or affected by overweight (BMI 25·0–29·9 kg/m^2^) or obesity (BMI ≥ 30·0 kg/m^2^). Due to the low number of underweight older adults (0·86 %, *n* 52), this group was combined with the normal-weight category for analysis. Weight loss information was self-reported via the home-administered weight history questionnaire, with a loss ≥ 10 % from the previous year’s weight considered significant.

Self-reported medical conditions were gathered through the question during the household interview, ‘*Has a doctor or other health professional ever told {you/Sample Person} that {you/s/he} …had [insert medical condition]?’* The medical conditions considered in this study included diabetes (Type 1, Type 2 or borderline), hypertension or elevated blood pressure and stroke. These conditions were specifically chosen due to their established associations with particular dietary habits^(38–40)^. Self-reported health was also assessed through the question on general health status (excellent to poor), with responses grouped into ‘excellent/very good/good’ *v*. ‘fair/poor’, following established classification methods^(41,42)^.

### Functional dentition

Dentition status was derived from the NHANES oral health exams conducted by dental professionals in the Mobile Examination Clinic. The examination assessed was based on tooth count, caries and sealants. Functional dentition was based on tooth count, excluding root fragments and third molars^(43)^. Participants were grouped as: ‘Complete or severe tooth loss’, (no natural teeth/implants or ≤ 8 teeth), ‘Lacking functional dentition’ (< 20 teeth) and ‘Functional dentition’ (≥ 20 of 28 teeth)^(44)^.

### Self-reported diet quality

Self-reported diet quality was assessed during the home interview. Responses of ‘fair’ or ‘poor’ to the question, ‘In general, how healthy is {your/his/her} overall diet? Would you say…’ were classified as poor diet quality, while ‘excellent’, ‘very good’, or ‘good’ were categorised as not poor.

### Food security status

Food security status was assessed using two validated scales. The first measured food security due to resource constraints using the 10-item Adult Food Security Survey Module from the NHANES Food Security Questionnaire, which measures experiences linked to insufficient financial resources for food acquisition over the preceding 12 months. Participants with 0–2 affirmative responses were considered food secure and 3–10 indicated food insecurity^(45)^. The second scale evaluated food insecurity due to physical functioning limitations, using the six-item Physical Food Security Scale for older adults^(46)^. It assessed difficulties in tasks like carrying heavy bags, preparing meals or shopping – that can affect one’s ability to shop for, prepare or consume food^(46)^. Individuals with three or more limitations were classified as having food insecurity due to food-related physical functioning limitations.

Participants were then categorised into four groups: Food secure on both scales, food insecure due to resource constraints only, food insecure due to physical functioning limitations only and food insecure on both scales.

### Deriving the dietary patterns

Dietary patterns were de novo derived using a data-driven cluster analysis of Day 1 dietary intake. Patterns were identified based on participants’ mean gram intake across 39 of USDA’s WWEIA food categories, and each cluster was named according to the food groups with the highest relative consumption. Examples include high consumption of 100 % fruit juices and soups, high intake of cured meats and sweet bakery products, or elevated intake of seafood and vegetables (Table 1).

**Table 1.** Mean intake (g) of selected What We Eat in America food categories, by dietary pattern cluster, among NHANES participants aged 60 years and older, NHANES 2013 to 2018^*^

	Juices, smoothies, grain drinks and soups (*n* 2680)		Cured meats, sandwiches and sweet bakery products (*n* 918)		Meats, alcohol and quick breads (*n* 557)		Cooked cereals and yogurt (*n* 547)		Seafood and vegetables (*n* 360)	
What We Eat in America food categories	Mean gram	se	Mean gram	se	Mean gram	se	Mean gram	se	Mean gram	se
Alcoholic beverages	75·8	6·3	87·3	10·1	**175·1**	**31·3** ^†^	64·4	12·5	146·9	19·6
Asian mixed dishes	15·9	2·4	6·6	3·1^‡^	5·5	3·9^‡^	**21·0**	**6·1** ^†^	6·3	2·4^‡^
Breads, rolls, tortillas	47·1	1·7	**49·2**	**3·0** ^†^	43·4	3·9	41·1	2·8	38·6	3·1
Candy	4·6	0·4	**13·4**	**1·9** ^†^	5·8	1·0	10·0	2·1	4·3	1·0
Cheese	15·5	0·9	14·4	2·7	12·5	1·8	**18·1**	**2·7** ^†^	11·3	1·5
Coffee and tea	411·4	12·2	**1019·9**	**39·3** ^†^	629·0	22·4	538·3	36·3	578·6	46·7
Cooked cereals	6·9	0·8	13·5	2·6	11·5	2·9	**139·6**	**14·5** ^†^	19·5	4·4
Cooked grains	21·4	2·0	7·6	1·6	13·8	2·6	27·0	4·2	**39·1**	**6·2** ^†^
Crackers	4·9	0·4	4·9	0·7	3·4	0·8	**5·0**	**1·0** ^†^	4·6	0·8
Cured meats and poultry	20·6	1·2	**44·7**	**3·9** ^†^	13·9	1·8	11·2	1·9	13·3	2·0
Diet beverages	69·6	6·0	**210·9**	**26·0** ^†^	81·5	12·1	101·7	25·7	52·7	10·6
Eggs and omelettes	26·1	1·4	17·4	1·9	27·0	2·5	12·6	2·0	**29·1**	**5·1** ^†^
Juice, 100 %	**66·3**	**4·1** ^†^	32·0	7·0	47·5	6·9	57·2	5·4	51·0	9·5
Grain-based mixed dishes	45·1	2·9	**88·7**	**11·9** ^†^	24·5	4·1	62·3	8·9	32·6	6·9
Meats	17·0	1·2	29·2	2·7	**290·6**	**11·7** ^†^	24·8	3·2	17·3	3·7
Mexican based mixed dishes	23·0	3·7	27·6	5·2	11·5	4·2^‡^	**28·5**	**7·5** ^†^	3·7	1·4^‡^
Milk	129·0	5·8	93·0	8·2	119·7	14·1	141·6	17·6	**145·0**	**23·0** ^†^
Nutritional beverages	11·0	2·4	2·9	0·9^‡^	4·3	1·9^‡^	**19·9**	**5·7** ^†^	13·5	4·2^‡^
Nuts and seeds	8·3	0·8	7·5	1·1	7·6	1·5	**27·0**	**4·7** ^†^	10·6	2·0
Other desserts	**33·5**	**2·3** ^†^	26·3	3·0	31·1	6·0	29·7	3·8	21·5	3·7
Pizza	12·1	2·3	17·0	3·5	5·4	2·8^‡^	**27·3**	**6·5** ^†^	0·8	0·4^‡^
Plant-based protein foods, excl nuts and seeds	15·0	1·4	10·9	2·1	**17·6**	**6·5** ^†^	14·6	3·7	13·2	4·8
Poultry	**51·7**	**3·0** ^†^	45·0	3·7	13·1	2·3	38·5	4·8	16·1	3·6
Quick breads and bread products	12·7	1·1	9·4	1·7	**31·1**	**4·4** ^†^	8·5	2·6	11·0	2·3
Ready-to-eat cereals	13·8	0·9	7·7	1·1	10·1	1·7	10·2	1·8	**14·7**	**2·8** ^**†,‡**^
Sandwiches	20·9	2·2	**87·9**	**8·2** ^†^	20·3	4·3	29·5	4·1	17·6	3·3
Sauces, oils, and condiments	31·6	1·2	**63·9**	**5·3†**	47·2	5·2	34·2	2·7	60·8	6·4
Savoury snacks	9·7	0·6	**16·4**	**1·8** ^†^	9·9	1·3	7·8	1·5	11·0	1·9
Seafood	5·4	0·6	3·3	0·7	2·4	0·9^‡^	19·0	4·8	**229·8**	**10·6** ^†^
Smoothies, grain drinks	**16·1**	**2·9** ^†^	1·3	0·5	5·2	2·4	10·5	3·7^‡^	12·9	5·2^‡^
Snack and meal bars	2·5	0·5	0·5	0·2	**2·9**	**1·7** ^**†,‡**^	2·7	0·7	1·8	0·9^‡^
Soups	**79·1**	**6·5** ^†^	40·1	8·0	46·0	13·3	58·8	8·2	33·9	10·0
Sugars	5·6	0·4	12·3	1·2	**18·0**	**2·8†**	7·4	1·0	7·5	1·3
Sweet bakery products	21·6	1·3	**101·7**	**6·6** ^†^	35·8	3·8	32·4	3·4	33·5	5·4
Sweetened beverages	105·1	6·3	186·6	20·9	**201·5**	**42·2** ^†^	67·2	7·4	101·3	26·4
Vegetables, excl white potatoes	86·7	4·0	60·9	4·5	109·9	17·4	69·1	6·6	**146·9**	**12·2** ^†^
White potatoes	25·1	1·7	**75·9**	**7·6** ^†^	69·2	8·2	22·8	4·9	69·8	12·1
Whole fruits	111·7	5·5	52·5	4·2	80·8	9·6	121·1	11·9	**122·0**	**10·0** ^†^
Yogurt	1·6	0·4	2·6	1·2	5·5	2·2	**127·4**	**9·2** ^†^	21·0	5·5

* Cured meats, sandwiches and sweet bakery products cluster consumed the most from the cured meats and poultry, sandwiches, sweet bakery products, coffee and tea, white potatoes and diet beverages. The fruit juices, smoothies, grain drinks and soups cluster consumed the most from 100 % juices, smoothies, grain drinks, soups and other desserts. The meats, alcohol and quick breads cluster consumed the most meats, added sugars, sweetened beverages, alcoholic beverages and quick breads and bread products. The seafood and vegetables cluster consumed the most of seafood, vegetables excluding white potatoes, whole fruits, ready-to-eat cereals and milk. The cooked cereals and yogurt cluster consumed the most of cooked cereals such as oatmeal and grits, yogurt and pizza.

† Mean intake of this food category was highest for this cluster (bold).

‡ Relative SE of the mean ≥ 30 %.

Each food and beverage reported by NHANES participants was coded using the Food and Nutrient Database for Dietary Studies 2013–2014, 2015–2016 and 2017–2018, which provides detailed food descriptions, ingredient lists, gram weights, and nutrient and energy values for each reported food and beverage^(47–49)^. These codes are linked to WWEIA classification, which organises foods commonly consumed in the American diet into 150 food groups^(50)^ based on similarity in nutrient content and usage. These categories were collapsed into 39 mutually exclusive food groups, excluding water and infant foods^(51,52)^. The 2017–2018 WWEIA classification was used to standardise categories across cycles.

Dietary patterns were identified using k-means cluster analysis using the FASTCLUS procedure in SAS software (version 9.4, SAS Institute Inc.)^(53)^. This approach assigns each individual to a single cluster based on the combined intake of all foods and nutrients consumed^(26)^. Standardised gram intakes (mean = 0, sd = 1) from the 39 WWEIA food groups were used to calculate Euclidean distances, preventing food groups with large variances from disproportionately influencing cluster assignments.

We evaluated several cluster solutions and selected a five-pattern solution based on the Pseudo-F statistic, cubic clustering criterion, R-squared plots and adequate sample sizes.

### Healthy Eating Index 2020

Total Healthy Eating Index 2020 (HEI-2020) and dietary component scores were estimated using the Population Ratio Method, following National Cancer Institute guidelines^(54)^. Food codes from Day 1 dietary recalls were grouped via the USDA’s Food Patterns Equivalents Database^(55)^ into 37 components, measured in cup and ounce equivalents^(55)^. Intakes and energy were aggregated and scored to reflect population-level dietary alignment with the 2020–2025 Dietary Guidelines for Americans^(56)^. A higher score indicates a greater alignment with the 2020–2025 Dietary Guidelines for Americans, including greater intake of beneficial food components such as total vegetables, greens and beans, total fruit, whole fruit, whole grains, dairy, total protein, seafood and plant protein, and a favourable ratio of fatty acids. A higher score also indicates a lower consumption of components to limit, such as sodium, refined grains, saturated fat and added sugars^(56)^.

### Total energy and protein intake

Data on total energy and protein intake were derived from the NHANES Dietary Interview component. The ‘Total Nutrient Intakes File’ was used, which provides detailed information for each participant on daily energy and nutrient consumption from food and beverages recorded during Day 1 of the dietary recall^(57)^.

### Statistical analysis

Percentages and means were estimated for the sociodemographic, economic and health characteristics of older adults by dietary pattern cluster. All estimates of proportions were evaluated using the NCHS data presentation standards for proportions^(58)^. Estimates not meeting the standards are designated by a footnote where applicable. In addition, mean estimates with relative se ≥ 30 % are flagged as unreliable^(58)^.

To compare differences between dietary pattern clusters, pairwise comparisons were conducted using contrasts (*t* statistics) in SUDAAN Proc Descript, to account for NHANES complex survey design. This approach accounts for sampling weights and design effects to ensure valid statistical inference. Differences between the dietary pattern clusters for the total and component HEI-2020 scores were also compared using contrasts in SUDAAN Proc Descript, and adjustments for multiple comparisons made using the Bonferroni test^(59)^. The threshold for statistical significance was set at *P* ≤ 0·05. All dietary analyses included dietary day 1 sample weights which account for oversampling, non-response, non-coverage and day of the week. se were estimated using Taylor series linearisation^(58,60)^. Analyses were conducted in SAS software (version 9.4, SAS Institute Inc)^(53)^, SUDAAN version 11.0 (RTI International)^(61)^ and STATA/BE software (version 17.0, Stata Corporation)^(62)^.

## Results

### Dietary patterns

Five distinct dietary patterns were identified among 5062 older adults, aged 60 years and older. To improve interpretability, each of the five dietary patterns was defined based on the food groups with the highest intake within that cluster. Detailed food group contributions are provided in Table 1.

The largest pattern, ‘juices, smoothies, grain drinks and soups’, comprising over half of older adults (*n* 2680), was characterised by high intakes of 100 % juices, soups, poultry, smoothies/grain drinks and other desserts (e.g. ice cream, sorbets, gelatins and puddings). The ‘cured meats, sandwiches and sweet bakery products’ cluster (*n* 918, approx. 18 %) had elevated consumption of cured meats and poultry, coffee/tea, sandwiches, sweet bakery products (cakes, cookies and pastries) diet beverages and white potatoes.

The ‘meats, alcohol and quick breads’ cluster (*n* 557; approx. 11 %) had the highest meat consumption, almost ten times higher than the next largest cluster, along with high intake of alcoholic beverages, and quick breads/bakery products (biscuits, muffins and pancakes). The ‘cooked cereals and yogurt’ (*n* 547; approx. 11 %) was marked by greater consumption of cooked cereals (oatmeal and grits), yogurt, cheese, nutritional beverages, as well as nuts and seeds and pizza. The smallest cluster, ‘seafood and vegetables’ cluster (*n* 360; 7 %), exhibited seafood intake almost 12 times higher than the next cluster, in addition to higher intakes of non-starchy vegetables, whole fruits, ready-to-eat cereals and milk.

### Sociodemographic, economic and health characteristics

Age and sex distributions differed significantly across dietary patterns (Table 2). Adults aged 60–69 years were more likely to follow the ‘cured meats, sandwiches and sweet bakery products’ (57·8 %) pattern compared with the ‘juices, smoothies, grain drinks and soups’ pattern (47·7 %). In contrast, those aged 70 years and above were more common in the ‘juices, smoothies, grain drinks and soups’ pattern (52·3 %). Males were more common in the ‘cured meats, sandwiches and sweet bakery products’ (54·9 %) and ‘meats, alcohol and quick breads’ (57·5 %) patterns, while females predominated in the ‘cooked cereals and yogurt’ (64·7 %) and ‘juices, smoothies, grain drinks and soups’ patterns (58·7 %).

**Table 2.** Sociodemographic characteristics of NHANES study participants, aged 60 years and over, by dietary pattern cluster, NHANES 2013 to 2018

	Total (*n* 5062)			Juices, smoothies, grain drinks and soups (*n* 2680)		Cured meats, sandwiches and sweet bakery products (*n* 918)		Meats, alcohol and quick breads (*n* 557)		Cooked cereals and yogurt (*n* 547)		Seafood and vegetables (*n* 360)	
Characteristics	*n*	Percent	95 % CI	Percent	95 % CI	Percent	95 % CI	Percent	95 % CI	Percent	95 % CI	Percent	95 % CI
Age category													
60–69 years	2659	52·6	50·2, 55·0	47·7^a^	44·4, 51·0	57·8^b^	52·4, 63·0	58·5^ab^	51·4, 65·2	54·0^ab^	45·2, 62·5	56·6^ab^	49·0, 63·9
70+ years	2403	47·4	45·0, 49·8	52·3^a^	49·0, 55·6	42·2^b^	37·0, 47·6	41·5^ab^	34·8, 48·6	46·0^ab^	37·5, 54·8	43·4^ab^	36·1, 51·0
Sex													
Male	2519	45·9	44·4, 47·4	41·4^a^	38·8, 44	54·9^b^	51·4, 58·3	57·5^b^	52·5, 62·4	35·3^a^	30·1, 40·9	46·8^ab^	39·6, 54
Female	2543	54·1	52·6, 55·6	58·7^a^	56·1, 61·2	45·1^b^	41·7, 48·6	42·5^b^	37·6, 47·5	64·7^a^	59·1, 69·9	53·3^ab^	46·0, 60·4
Race/ethnicity													
Non-Hispanic White	2248	76·4	72·7, 79·7	70·1^a^	66, 74	85·0^b^	81·4, 88·1	82·0^bc^	76·5, 86·4	79·4^bc^	72·7, 84·8	76·0^ac^	70·2, 81
Non-Hispanic Black	1085	8·6	6·9, 10·7	10·5^a^	8·6, 12·7	5·98^b^	4·3, 8·2	7·9^ab^	5·5, 11·2	6·1^b^	4·4, 8·5	10·3^ab^	7·1, 14·7
Hispanic	1194	8·3	6·5, 10·6	11·1^a^	8·6, 14·1	5·38^b^	3·8, 7·6	5·74^b^	4·1, 8·0	7·43^b^	5·1, 10·7	4·88^b^	3·5, 6·8
Non-Hispanic Asian & Other races	535	6·7	5·4, 8·4	8·4^a^	6·6, 10·5	3·6^b^	2·4, 5·4	4·4^bc^	2·7, 7	7·1^abc^	4·0, 12·1	8·9^ac^	3·5, 6·8
Marital status													
Married or living with a partner	2910	63·8	61·2, 66·3	61·0	57·7, 64·2	67·9	62·1, 73·2	63·8	56·4, 70·5	65·4	59·4, 70·9	67·0	56·8, 75·9
Divorced, separated, or never married	2148	36·2	33·7, 38·9	39·0	35·8, 42·3	32·1	26·8, 37·9	36·2	29·5, 43·6	34·6	29·1, 40·6	33·0	24·1, 43·2
Living arrangement													
Living alone	1247	23·4	21·5, 25·5	24·2	21·9, 26·7	20·8	16·8, 25·4	25·8	20·5, 32·0	24·0	19·5, 29·2	21·3	14·6, 30·0
Living with a spouse	2872	63·1	60·4, 65·7	60·2	56·8, 63·5	67·7	62, 73	63·4	56·2, 69·9	63·5	56·9, 69·6	66·8	56·6, 75·6
Living with someone other than a spouse	941	13·5	11·6, 15·7	15·6	12·9, 18·8	11·5	8·35, 15·7	10·8	7·8, 14·9	12·5	9·1, 17·1	11·9	7·0, 19·4
Educational level													
Below high school	1288	14·3	12·4, 16·5	17·5^a^	15·0, 20·3	11·0^b^	8·8, 13·8	11·3^b^	8·4, 15·0	12·0^b^	8·9, 16	13·1^ab^	8·8, 19·0
High school	1207	24·6	22·8, 26·4	25·1^ab^	22·4, 28	28·7^a^	24·9, 32·8	25·6^ab^	20·7, 31·2	17·7^b^	13·1, 23·5	18·3^ab^	12·2, 26·3
Above high school	2557	61·1	58·5, 63·7	57·4^a^	54·0, 60·8	60·3^ab^	55·8, 64·6	63·1^ab^	56·5, 69·3	70·3^b^	62·6, 77	68·7^ab^	59·8, 76·4
Family poverty-to-income ratio (PIR)													
At or below 185 % PIR	2050	26·4	23·5, 29·4	29·7^a^	26·5, 33·0	24·3^b^	20·0, 29·1	26·1^b^	21·2, 31·7	20·2^b^	14·9, 26·9	22·3^ab^	16·1, 30·1
Above 185 % PIR	2467	64·8	61·5, 67·9	59·8^a^	56·2, 63·3	68·2^b^	63·6, 72·6	68·2^b^	62·0, 73·8	70·8^b^	62·6, 77·8	70·4^ab^	62·1, 77·5

All pairwise tests performed using t statistic and levels for statistical testing were at the *P* < 0·05 significance level, and *P* values were adjusted using Bonferroni’s method of correction for multiple comparisons.

Groups that do not differ significantly share the same superscript letter.

Groups that are significantly different get different letters.

If a group overlap (i.e. not significantly different from two others), it may have multiple letters.

Racial/ethnic differences were also noted: Non-Hispanic White adults were highly represented in the ‘cured meats, sandwiches and sweet bakery products’ (85 %), while Hispanic adults were more common in the ‘juices, smoothies, grain drinks and soups’ pattern (11·1 %). The ‘Non-Hispanic Asian and Other’ group appeared less frequently in the ‘cured meats, sandwiches and sweet bakery products’ pattern (3·6 %) compared to the ‘juices, smoothies, grain drinks and soups’ (8·4 %) and the ‘seafood and vegetables’ patterns (8·9 %) (Table 2).

While marital status and living arrangement did not differ across patterns, disparities were observed in education and income. The ‘juices, smoothies, grain drinks and soups’ pattern had a higher proportion of older adults with less than a high school education and a poverty-to-income ratio below 185 %, compared with all patterns except the ‘seafood and vegetables’.

### Diet quality intake

HEI-2020 total scores varied significantly across dietary patterns (Table 3). The lowest scores were observed in the ‘cured meats, sandwiches and sweet bakery products’ pattern had the lowest HEI-2020 score (48·5 ± 1·0) followed by the ‘Meats, alcohol and quick breads’ pattern (58·3 ± 1·6). Both scores were significantly lower than those of both the ‘juices, smoothies, grain drinks and soups’ (66·0 ± 0·8) and the ‘cooked cereals and yogurt’ (72·3 ± 1·4) patterns. The ‘cooked cereals and yogurt’ pattern had the highest HEI-2020 score overall.

**Table 3.** Diet quality and intakes (energy and protein) in adults aged 60 and above by dietary patterns, NHANES 2013–2018

	Total (*n* 5062)		Juices, smoothies/grain drinks and soups (*n* 2680)		Cured meats, sandwiches and sweet bakery product (*n* 918)		Meats, alcohol and quick breads (*n* 557)		Cooked cereals and yogurt (*n* 547)		Seafood and vegetables (*n* 360)	
	Mean	se	Mean	se	Mean	se	Mean	se	Mean	se	Mean	se
Healthy Eating Index-2020 total score	62·6	0·6	66·0	0·8^a^	48·5	1·0^b^	58·3	1·6^bd^	72·3	1·4^c^	69·6	1·4^d^
	%	95 % CI	%	95 % CI	%	95 % CI	%	95 % CI	%	95 % CI	%	95 % CI
Poor/fair self-reported diet quality	19·8	17·7, 22·0	18·7^a^	16·6, 21·1	26·0^b^	22·0, 30·4	20·6^ab^	15·4, 27·0	14·6^a^	10·3, 20·3	15·1^a^	10·7, 20·8
	Mean	se	Mean	se	Mean	se	Mean	se	Mean	se	Mean	se
Total calories on Day 1 of 24-h recall	1913·0	18·4	1607·5	16·2^a^	2337·8	49·5^b^	2109·5	58·2^bc^	2031·4	61·9^c^	2085·5	63·4^c^
Total grams protein on Day 1 of 24-h recall	73·7	0·85	62·2	0·9^a^	79·2	1·7^b^	88·7	2·7^bc^	81·5	2·8^bc^	94·0	3·4^c^

All pairwise tests performed using *t* statistic and levels for statistical testing were at the *P* < 0·05 significance level, and *P* values were adjusted using Bonferroni’s method of correction for multiple comparisons.

Groups that do not differ significantly share the same superscript letter.

Groups that are significantly different get different letters.

If a group overlap (i.e. not significantly different from two others), it may have multiple letters.

**Figure 1:**
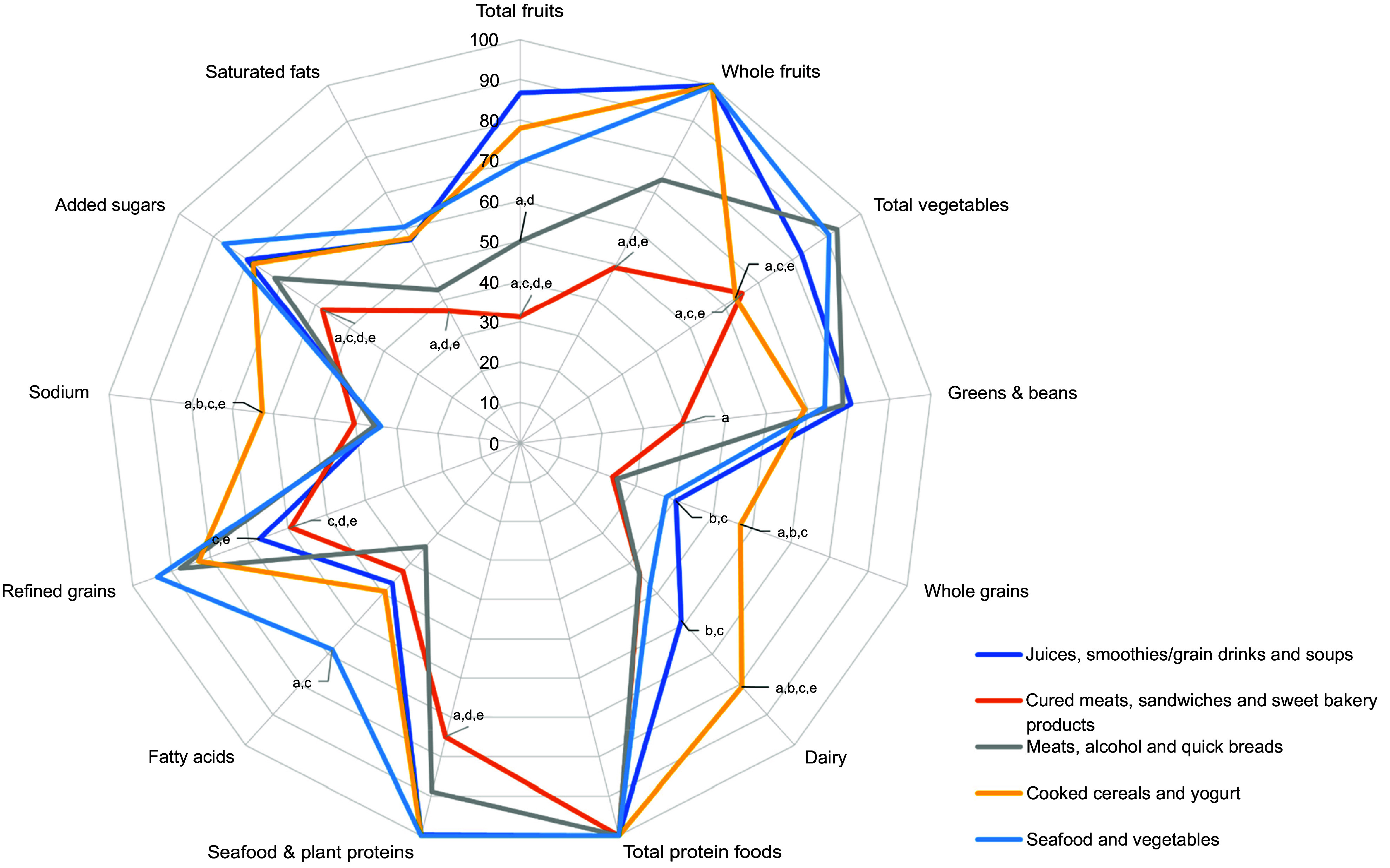
Comparison of HEI-2020 component scores among dietary patterns in older adults, NHANES 2013–2018. Note: Points nearer the centre represent lower values; those farther out represent higher values. ^a^ Significantly different than juices, smoothies, grain drinks and soups cluster *P* < 0·05. ^b^ Significantly different than cured meats, sandwiches and sweet bakery products cluster, *P* < 0·05. ^c^ Significantly different than meats, alcohol and quick breads cluster, *P* < 0·05. ^d^ Significantly different than cooked cereals and yogurt cluster, *P* < 0·05. ^e^ Significantly different than seafood and vegetables cluster, *P* < 0·05.

Figure 1 illustrates the distribution of the HEI-2020 component scores across dietary patterns. All patterns showed similar scores for total protein foods. The ‘cured meats, sandwiches and sweet bakery products’ pattern had the lowest adequacy scores (whole fruit, total fruit, greens and beans, seafood and plant proteins), and poor moderation scores, reflecting high intake of saturated fats, refined grains and added sugars. Along with the ‘meats, alcohol and quick breads’ pattern, it also had the lowest whole grain scores.

The ‘seafood and vegetables’ pattern scored significantly higher for fatty acid ratio score, indicating higher consumption of monounsaturated and polyunsaturated fats, in comparison to the ‘juices, smoothies, grain drinks and soups’ and the ‘cured meats, sandwiches and sweet bakery products’ patterns, The ‘cooked cereal and yogurt’ pattern had the highest dairy score and scored highest in whole grain intake, except for ‘seafood and vegetables.’

Older adults following the ‘cured meats, sandwiches and sweet bakery products’ pattern were more likely to rate their diet as fair or poor (26 %) compared to those in the ‘juices, smoothies, grain drinks and soups’ (18·7 %), ‘seafood and vegetables’ (15·1 %) and ‘cooked cereals and yogurt’ (14·6 %) patterns (Table 3).

Table 3 also presents Day 1 caloric and protein intake. The ‘juices, smoothies, grain drinks and soups’ pattern which had the lowest intake for both calories and proteins while the ‘cured meats, sandwiches and sweet bakery products’ had the highest caloric intake, significantly greater than all other patterns except ‘meats, alcohol and quick breads.’

### Health-related characteristics

Health characteristics also varied by dietary pattern (Table 4). The ‘cooked cereals and yogurt’ pattern had a higher proportion of older adults with a healthy BMI whereas the ‘cured meats, sandwiches and sweet bakery products’ pattern had the greatest proportion with obesity. Reports of significant weight loss over the past year did not differ across patterns.

**Table 4.** Health characteristics of NHANES study participants aged 60 and over, by dietary pattern cluster, NHANES 2013 to 2018

	Total (*n* 5062)			Juices, smoothies, grain drinks and soups (*n* 2680)		Cured meats, sandwiches and sweet bakery products (*n* 918)		Meats, alcohol and quick breads (*n* 557)		Cooked cereals and yogurt (*n* 547)		Seafood and vegetables (*n* 360)	
Characteristics	*n*	Percent	95 % CI	Percent	95 % CI	Percent	95 % CI	Percent	95 % CI	Percent	95 % CI	Percent	95 % CI
BMI (kg/m^2^)													
Under-or healthy weight (< 18·5–24·9)	1179	23·2	21·4, 25·2	24·7^ab^	22·0, 27·7	19·1^a^	15·8, 22·9	18·6^ab^	13·5, 25·2	29·4^b^	24·9, 34·4	23·1^ab^	15·9, 32·2
Overweight (25–29·9)	1812	35·9	33·8, 38·1	33·9	31·4, 36·6	35·4	30·3, 40·9	38·3	32·3, 44·7	40·4	34·4, 46·7	38·6	32·0, 45·8
Obese (≥ 30)	1989	40·9	38·2, 43·6	41·3^ab^	38·1, 44·6	45·5^a^	39·7, 51·4	43·1^ab^	36·6, 49·8	30·2^b^	24·0, 37·3	38·3^ab^	31·0, 46·2
Self-reported hypertension	3114	58·8	56·1, 61·5	61·3^a^	58·1, 64·5	55·9^ab^	51·0, 60·7	60·1^ab^	52·4, 67·4	50·2^b^	43·9, 56·6	64·1^ab^	53·7, 73·3
Self-reported diabetes	1354	22·0	20·4, 23·6	23·7	21·5, 26·1	21·0	18·3, 23·9	21·3	15·3, 28·7	20·1	15·5, 25·7	18·0	14·1, 22·8
Self-reported stroke	418	7·4	6·6, 8·2	8·5	7·2, 10	7·2	5·0, 10·2	5·4	3·6, 8·1	7·4	5·0, 11·0	3·9^*^	1·8, 8·1
Self-reported significant weight loss over one year (≥ 10 % body weight)	498	9·2	8·1, 10·3	9·2	7·8, 10·7	8·0	6·0, 10·5	10·4	5·8, 17·7	8·9	6·0, 13·2	11·0	6·4, 18·2
Smoking status													
Non-smoker	2510	50·3	48, 52·5	55·6^a^	53·2, 57·9	42·7^b^	37·9, 47·6	40·6^b^	34·7, 46·7	56·1^a^	49·2, 62·7	44·7^ab^	36·3, 53·5
Previous smoker	1890	38·9	37·0, 40·9	36·1	33·4, 38·9	40·5	36·9, 44·3	43·9	37·9, 50	37·3	30·6, 44·6	46·8	39·5, 54·3
Current smoker	654	10·8	9·5, 12·4	8·30^a^	6·9, 10	16·8^b^	13·9, 20·2	15·6^ab^	11·2, 21·2	6·6^c^	4·1, 10·5	8·4^ac^	4·7, 14·7
Poor/self-reported health	1458	19·8	17·6, 22·1	22·1^a^	19·6, 24·9	18^ab^	14·2, 22·6	19·8^ab^	17·6, 22·1	16·9^ab^	12·5, 22·4	14·3^b^	10·1, 19·8
Dentition status													
Complete or severe tooth loss (≤ 8 teeth)	1139	17·8	15·7, 20·2	17·3^ab^	15·0, 19·8	21·4^a^	17·4, 26·1	17·7^ab^	13·5, 22·9	13·0^b^	10, 16·7	19·1^ab^	12·9, 27·4
Lacking functional dentition (> 8 and < 20 teeth)	741	12·0	10·7, 13·5	12·3	10·7, 14·1	12·0	8·8, 16·1	13·9	10·1, 18·9	9·2	6·4, 13·1	11·9	7·9, 17·7
Functional dentition (≥ 20 teeth)	3182	70·2	67·6, 72·6	70·5^a^	67·6, 73·2	66·6^a^	61·9, 71·0	68·4^ab^	61·9, 74·1	77·9^b^	73·4, 81·8	69·0^ab^	60·3, 76·5

All pairwise tests performed using *t* statistic and levels for statistical testing were at the *P* < 0·05 significance level, and *P* values were adjusted using Bonferroni’s method of correction for multiple comparisons.

Groups that do not differ significantly share the same superscript letter.

Groups that are significantly different get different letters.

If a group overlap (i.e. not significantly different from two others), it may have multiple letters.

* Estimate has a relative CI width greater than 160 %.

The proportion of current smokers was markedly higher among individuals in the ‘cured meats, sandwiches and sweet bakery products’ pattern compared with most other patterns, except for those in the ‘meats, alcohol and quick breads’ (Table 4).

Self-reported hypertension was more common in the ‘juices, smoothies, grain drinks and soups’ (61·3 %) than in the ‘cooked cereal and yogurt’ pattern (50·2 %). In fact, more older adults in the ‘juices, smoothies, grain drinks and soups’ pattern rated their health as ‘poor’ (22·1 %), *v*. those in the ‘seafood and vegetables’ group (14·3 %).

Variation in dentition status across patterns was minimal though. ‘Cured meats, sandwiches and sweet bakery products’ pattern had the highest prevalence of complete or severe tooth loss and the lowest functional dentition, especially compared to the ‘cooked cereals and yogurt’ group.

### Food security status

Food security was most prevalent among individuals following the ‘seafood and vegetables’ pattern (85·2 %), significantly more than those in the ‘juices, smoothies, grain drinks and soups’ (74·1 %) (Table 5).

**Table 5.** Food security status, by dietary pattern clusters, among older adults 60 years and above, NHANES 2013 to 2018

	Total			Juices, smoothies/grain drinks and soups (*n* 2680)		Cured meats, sandwiches and sweet bakery products (*n* 918)		Meats and quick breads (*n* 557)		Cooked cereals and yogurt (*n* 547)		Seafood and vegetables (*n* 360)	
Food security status	*n*	Percent	95 % CI	Percent	95 % CI	Percent	95 % CI	Percent	95 % CI	Percent	95 % CI	Percent	95 % CI
High Food security	3005	77·0	74·6, 79·2	74·1^a^	71·1, 77·0	77·8^ab^	73·7, 81·4	79·2^ab^	73·3, 84·1	79·6^ab^	75·1, 83·5	85·2^b^	78·7, 90·0
Food insecurity – Resource-constraints	458	5·8	4·8, 6·9	6·7^a^	5·6, 8·0	5·5^ab^	3·6, 8·2	6·5^ab^	4·2, 10·1	3·5^b^	2·2, 5·7	2·9^b^	1·9, 4·4
Food insecurity – Physical limitations	663	13·5	12·2, 15·0	14·7	12·6, 7·0	12.5	9·7, 15·9	11·6	8·0, 16·5	14·5	11·2, 18·4	11·4	7, 17·9
Food insecurity – Economic and Physical	280	3·7	3·0, 4·5	4·5^a^	3·4, 6·0	4·3^a^	3·1, 6·0	2·7^a^	1·5, 4·6	2·4^ab^	1·4, 4·2	0·5^b^	0·2, 1·4
*n*	4406			2321		816		491		464		314	

Groups that do not differ significantly share the same superscript letter.

Groups that are significantly different get different letters.

If a group overlap (i.e. not significantly different from two others), it may have multiple letters.

Food insecurity due to resource constraints was highest in the ‘juices, smoothies, grain drinks and soups’ pattern (6·7 %), exceeding rates in the ‘cooked cereals and yogurt’ (3·5 %) and ‘seafood and vegetables’ (2·9 %) patterns.

Food insecurity linked to physical functioning limitations was also most common among those in the ‘juices, smoothies, grain drinks and soups’ (14·7 %), and ‘cooked cereals and yogurt’ (14·5 %) patterns though differences were not statistically significant. Food insecurity due to both resource constraints and physical functioning limitations was least prevalent among individuals in the ‘seafood and vegetables’ patterns (0·5 %), significantly lower than in all patterns except for ‘cooked cereals and yogurt’ (2·4 %).

## Discussion

Using national representative NHANES data and cluster analysis, we identified five distinct dietary patterns. Two patterns that may be unique to older adults are the ‘juices, smoothies, grain drinks and soups’ and the ‘cooked cereals and yogurt’, which are characterised by softer foods and liquids. This suggests a preference for easily digestible options and lower food preparation, which may appeal to individuals with functional limitations. Despite their shared textural qualities, these dietary patterns differ significantly in nutritional quality, sociodemographic composition and health status. The ‘cooked cereals and yogurt’ pattern achieved the highest HEI-2020 score, reflecting greater alignment with dietary guidelines, particularly through higher intake of dairy, whole grains and lower intake of sodium and refined grains; foods associated with improved physical functioning, bone health and cognitive resilience in older adults.

In contrast, the highly prevalent ‘juices, smoothies, grain drinks and soups’ pattern had substantially lower energy and protein intake which may exacerbate age-related nutritional risks^(63)^. Protein insufficiency accelerates muscle loss and functional decline, potentially increasing the prevalence of sarcopenia and limiting mobility. Collectively, these results suggest that while soft diets may be appropriate for some older adults, they also present risks when they lack adequate protein and energy density. Thus, identifying strategies to ensure that older adults can maintain soft-food diets while meeting nutritional needs is a priority for clinicians and public health practitioners.

We expected the softer foods dietary patterns to be more common among individuals with poor dentition, swallowing disorders or specific medical conditions^(64–66)^. Surprisingly, dentition status did not differ significantly across patterns. In fact, the ‘cooked cereals and yogurt’ pattern included a higher proportion of older adults with functional dentition than the ‘juices, smoothies, grain drinks and soups.’ These results suggest that other factors such as education, income and nutrition knowledge may drive healthier food choices even among those preferring soft-textured diets. However, as expected, those in the ‘juices, smoothies, grain drinks and soups’ cluster had a higher proportion of comorbidities such as hypertension and poorer self-rated health. Additionally, the elevated prevalence of food insecurity particularly when related to economic constraints or combined with physical limitations aligns with prior studies showing that chronic conditions often prompt dietary modifications toward lower sodium, lower energy or softer foods^(67,68)^.

The three additional dietary patterns were also distinct in their nutritional and sociodemographic profiles and closely align with those observed in previous research involving older adults. Two patterns the ‘cured meats, sandwiches and sweet bakery products’ and the ‘meats, alcohol and quick breads’ exhibit similarities with ‘Western’-style eating patterns, reflecting eating habits characterised by frequent consumption of processed or fast foods. Such dietary behaviours are strongly associated with elevated dietary sodium intake, hypertension, obesity and chronic metabolic diseases. These patterns also exhibit similarities in sociodemographic and health-related traits, such as a predominance of male and non-Hispanic White older adults, when compared to other dietary patterns.

These two patterns had the lowest HEI-2020 scores, were most prevalent among the younger age groups (aged 60–69 years) and were significantly associated with a higher proportion of current smokers. These findings align with prior research linking cigarette smoking to poor nutrient profiles, such as high consumption of saturated fats and alcohol, alongside lower intakes of polyunsaturated fats^(69,70)^. The ‘cured meats, sandwiches and sweet bakery products’ demonstrated the poorest adherence to the Dietary Guidelines for Americans 2020–2025, as reflected by its lowest total HEI-2020 score across all patterns. Older adults in this group were more likely to rate their diet quality as poor, reinforcing the reliability of the single-item self-rated diet quality measure^(71)^. This group also had a higher prevalence of obesity compared to those adhering to the ‘cooked cereal and yogurt pattern’. Both patterns also showed a greater prevalence of food insecurity due to coexisting economic constraints and physical functional limitations compared to individuals in the ‘seafood and vegetables’ cluster, supporting existing research that links food insecurity with unhealthy dietary patterns^(11)^. Research has shown that food-insecure people often replace higher-quality, nutrient-rich foods, such as fruits, vegetables and lean proteins, with more affordable, readily available and energy-dense alternatives like carbohydrates and processed foods^(22,72–74)^.

In contrast, the ‘seafood and vegetables’ pattern, which was followed by the fewest participants, resembled a Mediterranean-style dietary pattern, characterised by high intake of fruits, vegetables, nuts, legumes and seafood. This pattern is consistent with better adherence to the Dietary Guidelines for Americans and is representative of healthful eating in later life. Its HEI-2020 total score was the second highest, following the ‘cooked cereals and yogurt’ group, likely due to shared characteristics such as socioeconomic status, food security and educational attainment. These findings are consistent with prior research showing that higher food security is associated with better diet quality and adherence to dietary guidelines^(10)^.

Importantly, nearly one-third of older adults in this study followed dietary patterns with low alignment to the Dietary Guidelines for Americans. Given the strong evidence linking such patterns to chronic disease, inflammation and diminished quality of life^(75,76)^, these findings highlight the need for interventions that address both behavioural and structural barriers to healthy eating among older adults. Nutrition education and behavioural change programmes that promote healthier eating and raise awareness of the risks associated with excessive saturated fat and alcohol intake should especially target younger adults who are more likely to consume processed food. These programmes should be culturally tailored, particularly for Hispanic and non-Hispanic Black older adults who were disproportionately represented in lower-quality dietary patterns.

Programmes should also address barriers related to mobility, food preparation and access. The findings can inform policies aimed at strengthening subsidies for nutrient-dense foods, expanding home delivery or congregate meal programmes and promoting community-based initiatives that improve access to affordable and healthy options. Nutrition policy may also need to better account for functional limitations. Screening for physical functioning barriers during healthcare or nutrition assessments may help identify individuals at risk for low-quality diets. Policies supporting convenient yet nutritious foods, such as pre-cut vegetables, fortified soups and ready-to-heat whole grains, may also help older adults who rely on soft or easy-to-prepare foods.

Clinicians should monitor older adults who consume soft-textured diets for signs of inadequate caloric or protein intake and provide counselling on nutrient-dense soft foods (e.g. yogurt, pureed beans and blended vegetable soups). Public health planners should consider the high proportion of older adults following low-quality patterns when designing dietary interventions for aging populations. Importantly, the substantial proportion of older adults exhibiting unhealthy eating behaviours reflects a significant public health concern highlighting the importance for interventions that address both behavioural and structural barriers to improving dietary quality.

This study has several limitations and strengths. These include the reliance on self-reported dietary intake, which can underreport energy intake^(77,78)^. However, the NHANES dietary data collection uses the USDA Automated Multiple-Pass Method^(32)^, widely regarded as the gold standard for both self-reported and proxy-reported 24-hour recalls. This method enhances accuracy by prompting respondents to recall specific foods that might otherwise be overlooked. Another limitation is the cross-sectional design of NHANES, which precludes the establishment of causal relationships between lifestyle factors and dietary intake. Finally, we did not use the NHANES data released beyond 2018 because in the following years, the survey did not include the variables that we used to generate the six-item Physical Food Security Scale. Therefore, due to differences in questionnaire design and the lack of direct comparability, we relied on the consistent PFQ data from 2013–2018.

Despite these limitations, the study has several strengths. It uses nationally representative data, focuses on older adults – an understudied population, incorporates both economic and physical food insecurity measures, uses the most recent HEI-2020 scoring system and uses the population ratio method which provides a robust estimate of usual mean intakes. Finally, this research responds to the call from the Dietary Guidelines for Americans 2020–2025^(79)^ for expanded investigation into dietary patterns. This study contributes meaningfully to the broader discourse on nutrition and public health.
